# Supplementary material for: The force–length–velocity potential of the human soleus muscle is related to the energetic cost of running
Source: Proc Biol Sci. 2019 Dec 18;286(1917):20192560. doi: 10.1098/rspb.2019.2560 (PMC6939913; doi:10.1098/rspb.2019.2560)
Supplement: Methods supplementary from The force-length-velocity potential of the human soleus muscle is related to the energetic cost of running [file rspb20192560supp1.pdf]

Supplementary information on methods

"The force-length-velocity potential of the human soleus muscle is related to the energetic cost of running"

by Sebastian Bohm, Falk Mersmann, Alessandro Santuz & Adamantios Arampatzis

Journal: Proceedings of the Royal Society B

DOI: http://dx.doi.org/10.1098/rspb.2019.2560

Determination of the ankle joint moments during MVC

The resultant moments at the ankle joint were calculated by means of an established inverse dynamics approach [1], which takes the effects of gravitational and passive moments and any misalignment between ankle joint axis and dynamometer axis into account. The required kinematic data were recorded during the MVCs on the basis of anatomically referenced reflective markers (medial and lateral malleoli and epicondyle, calcaneal tuberosity, 2nd metatarsal and greater trochanter) using a Vicon motion capture system (Version 1.8, Vicon Motion Systems, Oxford, UK). The ankle joint angle-specific moments due to gravity and passive moments were measured during an additional ankle joint rotation driven by the dynamometer at 5 °/s with the participants completely relaxed. Thus, moments due to gravity and passive moments in a certain joint angle were attributed to the measured moment during the MVCs in the same joint angle configuration [1]. Furthermore, the contribution of the antagonistic muscles to the different measured ankle joint moments [2] was considered by establishing an individual relationship of EMG amplitude of the tibialis anterior muscle, agonistic moment as well as ankle joint angle. For this reason, EMG activity was measured at rest and during two submaximal isometric dorsiflexion contractions that displayed slightly lower and higher EMG magnitude as during the maximum plantar flexions [2] in three different joint angles (i.e. dorsiflexion, neutral position and plantar flexion) within the assessed range of motion. The relationship was described by the following regression equation:

$$M_{coact} = EMG_{tib.\ ant.} \cdot (a + b \cdot \alpha_{ankle} + c \cdot {\alpha_{ankle}}^2) \qquad \text{(eq. 1)}$$

where M_{coact} is the antagonistic joint moment during the maximum plantar flexion, $EMG_{tib.\ ant.}$ is the respective tibialis anterior EMG activity during the MVCs, α_{ankle} the ankle joint angle measured via the Vicon system and *a*, *b* and *c* the individual regression coefficients. This means that for each joint angle the relationship between moment and EMG activity was assumed to be linear because of the small differences of the EMG magnitude of the two submaximal isometric dorsal flexion contractions [2]. The joint angle-moment relationship presented by the three different measured angles was than formulated by a quadratic function to account for the force-length dependence of the muscle. EMG activity of the tibialis anterior and soleus muscle was measured using a wireless EMG system (Myon m320RX, Myon AG, Baar, Switzerland) and two bipolar surface electrodes (2 cm inter-electrode distance) that were placed on the muscle at an acquisition frequency of 1000 Hz, synchronized with the kinematic data.

Determination of the Achilles tendon lever arm

The Achilles tendon lever arm was individually determined by means of the tendon excursion method [3,4]. In this method, the lever arm of the Achilles tendon is calculated as the ratio of the m. gastrocnemius medialis myotendinous junction displacement obtained by ultrasonography to the

corresponding angular excursion of the ankle joint during a passive joint rotation by the dynamometer (5 °/s). The lever arm values were further corrected for the alignment of the tendon occurring during contractions using the factor provided by Maganaris et al. (1998) [5].

Fascicle length determination from the ultrasound images

The procedure included an approximation of the deeper and upper aponeurosis by a best linear fit through three manually placed and frame-by-frame adjusted marks. By means of the *bwtraceboundary* function of the *Matlab Image Processing toolbox* the algorithm then identified the shape and orientation of image brightness features between both aponeuroses in each frame, which are indicative for the hyperechoic perimysial connective tissue parts aligned with the muscle fascicles (fig. 1A in main manuscript). The feature identification criteria were set to: minimal length of 23 pixels (i.e. 0.4 cm, from the bottom left to the top right), area to length ratio of 8.5, angle between feature and deeper aponeurosis between 10° and 80° and 80% of the pixels on a line between the start and end point of a feature had to be white [6]. Every frame was visually controlled for adequate feature placement and manually corrected if necessary. Based on the identified features, a linear averaged reference fascicle was calculated (fig. 1A in main manuscript). Reliability of the tracking approach was confirmed and reported in two previous studies [6,7].

EMG processing

Raw EMG signals from the running and MVC trials were processed by a fourth-order high-pass Butterworth zero-phase filter with a 50 Hz cut-off frequency then a full-wave rectification and a low-pass zero-phase filter with a 20 Hz cut-off frequency for creating a linear envelope of the signal [8,9].

References

1. Arampatzis A, Morey-Klapsing G, Karamanidis K, DeMonte G, Stafilidis S, Brüggemann G-P. 2005 Differences between measured and resultant joint moments during isometric contractions at the ankle joint. J. Biomech. 38, 885–892. (doi:10.1016/j.jbiomech.2004.04.027)
2. Mademli L, Arampatzis A, Morey-Klapsing G, Brüggemann G-P. 2004 Effect of ankle joint position and electrode placement on the estimation of the antagonistic moment during maximal plantarflexion. J. Electromyogr. Kinesiol. 14, 591–597. (doi:10.1016/j.jelekin.2004.03.006)
3. An KN, Takahashi K, Harrigan TP, Chao EY. 1984 Determination of muscle orientations and moment arms. J. Biomech. Eng. 106, 280–282.
4. Fath F, Blazevich AJ, Waugh CM, Miller SC, Korff T. 2010 Direct comparison of in vivo Achilles tendon moment arms obtained from ultrasound and MR scans. J. Appl. Physiol. Bethesda Md 1985 109, 1644–1652. (doi:10.1152/japplphysiol.00656.2010)
5. Maganaris CN, Baltzopoulos V, Sargeant AJ. 1998 Changes in Achilles tendon moment arm from rest to maximum isometric plantarflexion: in vivo observations in man. J. Physiol. 510, 977–985. (doi:10.1111/j.1469-7793.1998.977bj.x)
6. Marzilger R, Legerlotz K, Panteli C, Bohm S, Arampatzis A. 2018 Reliability of a semi-automated algorithm for the vastus lateralis muscle architecture measurement based on ultrasound images. Eur. J. Appl. Physiol. 118, 291–301. (doi:10.1007/s00421-017-3769-8)
7. Bohm S, Marzilger R, Mersmann F, Santuz A, Arampatzis A. 2018 Operating length and velocity of human vastus lateralis muscle during walking and running. Sci. Rep. 8, 5066. (doi:10.1038/s41598-018-23376-5)

8. Nikolaidou ME, Marzilger R, Bohm S, Mersmann F, Arampatzis A. 2017 Operating length and velocity of human M. vastus lateralis fascicles during vertical jumping. R. Soc. Open Sci. 4, 170185. (doi:10.1098/rsos.170185)

9. Santuz A, Ekizos A, Janshen L, Baltzopoulos V, Arampatzis A. 2017 On the Methodological Implications of Extracting Muscle Synergies from Human Locomotion. Int. J. Neural Syst. 27, 1750007. (doi:10.1142/S0129065717500071)
